# Intermolecular Interactions as a Measure of Dapsone Solubility in Neat Solvents and Binary Solvent Mixtures

**DOI:** 10.3390/ma16186336

**Published:** 2023-09-21

**Authors:** Piotr Cysewski, Maciej Przybyłek, Tomasz Jeliński

**Affiliations:** Department of Physical Chemistry, Pharmacy Faculty, Collegium Medicum of Bydgoszcz, Nicolaus Copernicus University in Toruń, Kurpińskiego 5, 85-096 Bydgoszcz, Poland; m.przybylek@cm.umk.pl (M.P.); tomasz.jelinski@cm.umk.pl (T.J.)

**Keywords:** dapsone, solubility, machine learning, intermolecular interactions, affinity, COSMO-RS, neat solvents, binary mixtures

## Abstract

Dapsone is an effective antibacterial drug used to treat a variety of conditions. However, the aqueous solubility of this drug is limited, as is its permeability. This study expands the available solubility data pool for dapsone by measuring its solubility in several pure organic solvents: N-methyl-2-pyrrolidone (CAS: 872-50-4), dimethyl sulfoxide (CAS: 67-68-5), 4-formylmorpholine (CAS: 4394-85-8), tetraethylene pentamine (CAS: 112-57-2), and diethylene glycol bis(3-aminopropyl) ether (CAS: 4246-51-9). Furthermore, the study proposes the use of intermolecular interactions as molecular descriptors to predict the solubility of dapsone in neat solvents and binary mixtures using machine learning models. An ensemble of regressors was used, including support vector machines, random forests, gradient boosting, and neural networks. Affinities of dapsone to solvent molecules were calculated using COSMO-RS and used as input for model training. Due to the polymorphic nature of dapsone, fusion data are not available, which prohibits the direct use of COSMO-RS for solubility calculations. Therefore, a consonance solvent approach was tested, which allows an indirect estimation of the fusion properties. Unfortunately, the resulting accuracy is unsatisfactory. In contrast, the developed regressors showed high predictive potential. This work documents that intermolecular interactions characterized by solute–solvent contacts can be considered valuable molecular descriptors for solubility modeling and that the wealth of encoded information is sufficient for solubility predictions for new systems, including those for which experimental measurements of thermodynamic properties are unavailable.

## 1. Introduction

Dapsone (DAP, CAS number: 80–08-0, 4,4’-diaminodiphenylsulfone, C_12_H_12_N_2_O_2_S) is an effective antibacterial drug, being a part of the sulphone class, used to treat a variety of conditions [1]. It is used in both topical and systemic forms to treat a variety of conditions, including leprosy, malaria, dermatitis herpetiformis, acne, and diseases associated with AIDS [2,3,4,5]. Dapsone works by inhibiting the production of folic acid through the competitive inhibition of dihydropteroate synthetase, which ultimately disrupts the synthesis of nucleic acids essential for bacterial survival and reproduction, accounting for DAP’s antibacterial activity [2,6]. Furthermore, the anti-inflammatory effect of dapsone can also be attributed to its ability to regulate the production of cytokines [7,8]. Dapsone also has antioxidant properties by limiting the generation of ROS and superoxide radicals. This is achieved by binding to NADPH oxidase [9]. The liver metabolizes dapsone through acetylation and hydroxylation, with primary elimination occurring via urine [10,11]. There are some negative effects associated with dapsone, which include, among others, some hematological problems and peripheral neuropathy, with the potential for hypersensitivity syndrome and a life-threatening drug reaction [12]. Due to its low water solubility and permeability, dapsone falls under class II of the Biopharmaceutics Classification System (BCS) [13]. Consequently, transdermal administration is favored over oral ingestion [14]. These limitations prompted various approaches to address dapsone’s poor solubility and limited bioavailability [15,16]. Notably, an interesting way to enhance the dissolution behavior of dapsone is by using deep eutectic solvents (DESs) [17].

Solubility stands as a fundamental physicochemical property with significant implications in both theoretical and practical aspects [18,19]. Beyond its clear influence on bioavailability, solubility is an important technological factor [20]. This is understandable since, as estimated, most of the chemical compounds used in drug manufacturing are solvents [21,22]. For this reason, theoretical solubility prediction methods are useful tools that support the selection of an optimal solvent or solvent mixtures for technological purposes, including in the pharmaceutical industry [23,24,25]. First principle methods utilizing quantum chemistry computations augmented with statistical analysis, such as conductor-like screening model for realistic solvents (COSMO-RS) [26,27,28], deserve special attention. In general, this method has been found to be successful in modeling various pharmaceutically relevant properties and characteristics, including solubility [29,30,31,32], partition coefficients [33,34,35], acid–base properties [36,37,38], and co-crystallization abilities [27,39,40,41,42,43]. In recent years, the application of neural networks, including deep learning, to solubility modeling has increased [44,45,46]. As it was established in the previous works [47,48,49,50], combining COSMO-RS with machine learning methods can achieve high prediction accuracy. The purpose of this paper is threefold. Firstly, the available pool of dapsone solubility–temperature profiles is carefully cured and augmented with new measurements in neat solvents, such as N-methyl-2-pyrrolidone, dimethyl sulfoxide, 4-formylmorpholine, tetraethylene-pentamine, and diethylene glycol bis(3-aminopropyl) ether, for extending the diversity of the dissolution media. Secondly, the most probable solute–solvent contacts are characterized on an advanced ab initio level. Finally, the methods of solubility screening are provided, addressing the fundamental problem of the lack of the fusion thermodynamic properties of dapsone.

## 2. Materials and Methods

### 2.1. Materials

Dapsone (DAP, CAS Number: 80–08-0) was obtained from Sigma Aldrich (Saint Louis, MO, USA) at a purity of over 99%. The solvents used in the study, namely N-methyl-2-pyrrolidone (NMP, CAS Number: 872-50-4), dimethyl sulfoxide (DMSO, CAS Number: 67-68-5), 4-formylmorpholine (4FM, CAS Number: 4394-85-8), tetraethylenepentamine (TEPA, CAS Number: 112-57-2), and diethylene glycol bis(3-aminopropyl) ether (B3APE, CAS Number: 4246-51-9). Moreover, methanol (CAS Number: 67-56-1) was used as an auxiliary solvent for dilution purposes. All solvents were similarly purchased from Sigma Aldrich, and their purity was no less than 99%.

### 2.2. Solubility Measurements

In this study, the shake-flak method of solubility determination was applied. The method has been used and validated in previous work by our group on various pharmaceuticals or drug-like compounds, including sulfa drugs (sulfanilamide [51], sulfamethizole [49]), amides (nicotinamide [52], benzamide, salicylamide, and ethenzamide [32]), acetanilide derivatives (phenacetin [53]), and nutraceuticals (coumarin [54]). Based on this protocol, the solubility determination of dapsone was preceded by the construction of a calibration curve. In the first step, a stock solution of DAP was prepared by dissolving 2.7 mg of dapsone in methanol in a 100 mL volumetric flask. Afterward, several dilutions were made in order to obtain solutions with decreasing solute concentrations in the range from 0.00135 mg/mL to 0.0135 mg/mL with a total of 14 points characterized by varying concentrations. The solutions prepared in this way were subjected to spectrophotometric measurements using an A360 spectrophotometer from AOE Instruments (Shanghai, China). The maximum absorbance of the samples corresponded to the wavelength of 295 nm. Three separate curves were prepared, and the final one was the result of their averaging. The range of mean absorbance values was from 0.159 to 1.540. The linear regression equation of the final curve was A = 113.8664∙C + 0.0015 (A—absorbance, C—concentration expressed in mg/mL), and the determination coefficient indicated a high degree of linearity with R^2^ = 0.999. The calculated limits of detection and quantification were found to be LOD = 3.71∙10^−4^ mg/mL and LOQ = 1.11∙10^−3^ mg/mL, respectively.

To evaluate the solubility of dapsone in different solvents, its excess amounts were introduced into test tubes containing a specific solvent. The resulting saturated solutions were then placed in an Orbital Shaker Incubator ES-20/60 from Biosan (Riga, Latvia) and subjected to incubation at different temperatures over a 24 h period. Four temperature increments were used for this incubation, ranging from 298.15 K to 313.15 K at 5 K intervals. The temperature of the incubator remained within 0.1 degrees, and fluctuations within 0.5 degrees were observed throughout the 24 h cycle. Simultaneously, the samples were agitated at a rate of 60 revolutions per minute. Subsequently, the samples were filtrated using syringes equipped with PTFE filters featuring a pore size of 0.22 µm. To prevent any precipitation arising from temperature disparities between the solutions and the apparatus, all elements, including test tubes, pipette tips, syringes, and filters, underwent preheating. These items were placed within the same incubator as the samples, attaining the same temperature prior to manipulation. This step was particularly vital when dealing with higher temperatures. Following filtration, minor aliquots of the obtained filtrate were diluted within test tubes containing methanol and subjected to spectrophotometric measurement. The density of each solution was determined by weighing a 1 mL volume within 10 mL volumetric flasks, utilizing an Eppendorf Reference 2 pipette (Hamburg, Germany) with a systematic error of 6 μL. Additionally, the RADWAG AS 110 R2.PLUS analytical balance (Radom, Poland) with a precision of 0.1 mg was employed for this purpose. The process of solubility determination utilized the same spectrophotometer as was the case for the calibration curve preparation. Spectral data were captured within the wavelength span of 190 nm to 400 nm, maintaining a resolution of 1 nm. Throughout these procedures, methanol served both as the diluent for the samples and for the initial calibration of the spectrophotometer. The analytical wavelength was specifically set at 295 nm, and the absorbance at this wavelength was used to quantify the DAP concentration present in the samples, subsequently enabling the calculation of its mole fractions. Three distinct measurements were undertaken, with the resulting values being averaged for increased reliability.

### 2.3. Solubility Dataset Curation

Dapsone solubility values have been reported for twelve pure solvents [55] and four binary mixtures [56] as inferred from an extensive literature search. Unfortunately, there are two problems with this collection. First, there are some inconsistencies in the solubility values, which require careful consideration prior to nonlinear model formulation. Second, the solvent space is rather limited with insufficient diversity of solvent properties. In fact, DAP solubility has been collected mainly in polar protic solvents such as alcohols and polar aprotic solvents such as esters or acetone. The first aspect will be addressed by data curation and the latter by expanding the pool of solubility data with new measurements.

Although the diversities of reported DAP solubility in different sources are quite modest, they are still non-trivial and require standardization. This was carried out here by using the three-parameter van’t Hoff equation, ln(x^vHF^) = A + B·T^−1^ + C·T^−2^ [57,58,59,60,61,62]. This simple polynomial extension of the original model accounts for a non-constant enthalpy value [62] of the solubilization process. This simple model does not require any additional information other than solubility data and is very accurate in back-calculations, provided that the A, B, and C parameters are optimized. This was performed by using gradient optimization to minimize the values of RMSD (root means square deviation) using the solver implemented in MS Excel. The back-computed solubility values for the experimental temperatures were used for model building. The characteristics of the whole curated solubility dataset are provided in the supporting materials (see Section S1 in File S1) together with the obtained consensus values used for model formulation (see column “log(x^CONS^)” in the “data” spreadsheet of the File S2). The values of solubility data in binary mixtures were used as reported in the literature [56] except for neat solvents, which were replaced with consensus data.

### 2.4. Computations Protocol

#### 2.4.1. COSMO-RS Solubility Computations

The COSMO-RS approach [26,27,28] implemented in BIOVIA COSMOtherm 2021 (build: 21.0.0 [d1b290c105]) [63] was used for dapsone solubility computations. Since the iterative procedure occasionally fails in solubility computations, especially for cases with high solubility, the complete solution of solid–liquid equilibrium (SLE) was used by toggling the SLESOL option. Furthermore, since COSMOtherm can only treat liquids, a hypothetical subcooled liquid state is postulated in the case of solid solubility, and the thermodynamic contribution of an ordered solid transition to the random particle distribution in the subcooled liquid requires the provision of the values of the Gibbs free energy of fusion. Since neither the melting temperature, nor the heat of fusion, nor the change in heat capacity upon melting is experimentally undeterminable, the reference solvent approach was used. The main advantage of this practical approach is the ability to evaluate fusion thermodynamics from the provided experimental solubility in another solvent. Unfortunately, the calculated solubility values are strongly influenced by the choice of reference data. This is due to the fact that the errors of COMSO-RS are of similar magnitude for compounds with comparable structures. Therefore, screening is essential to optimize the number and type of reference solvents. This set of best-selected reference solvents is called consonance solvents and can minimize the overall error of solubility calculations [64]. For an adequate representation of the structure of dapsone and all solvents, the sets of relevant conformations were generated using COSMOconf 4.2 [65] and optimized with the aid of TURBOMOLE version TURBOMOLE V7-5-1 (V7-5-1 23 Dez 2020, Dassault Systèmes: Vélizy-Villacoublay, France,) [66] interfaced with BIOVIA TmoleX version 21.0.1 [67] as a default engine for geometries optimization. The obtained conformers used further for characteristics of bulk systems had their geometries fully optimized using BP functional and TZVP basis sets. All structures were generated both in the gas phase and including environmental effects via the COSMO-RS solvation model [28]. For solubility computations, the TZVPD-FINE level was used, which corresponds to single-point calculations with the TZVPD basis set and the same density functional based on previously generated geometries. The BP_TZVPD_FINE_21.ctd parameter set was used for all physicochemical property computations using COSMOtherm.

#### 2.4.2. Affinity Characteristic of Solute–Solvent Systems

The conformational screening of solute–solute and solute–solvent bi-molecular systems was performed prior to affinity characteristics. The methodology is consistent with previously published work [50,68]; hence, only a brief description is given below. This step was aimed at finding geometries of the most probable clusters. For this purpose, the COSMOtherm program facilities were used by calculating the contact statistics based on the probability of interactions between molecule surface segments. Practically, it is performed by using the “CONTACT={1 2} ssc_probability ssc_weak ssc_ang=15.0” command and automatic generation of contacts by alteration of the mutual orientation of the two contacting molecules with a 15° step rotation interval. Weak interactions are also included in the probability statistics as evidenced by the above prompt. Usually, this leads to a quite large number of potential structures whose geometries are far from optimal. Hence, the structure optimizations were performed using RI-DFT BP86 (B88-VWN-P86). In the final step, the number of pairs was reduced by comparing their energy values and RMSD values after cluster overlapping. Highly similar clusters and the ones exceeding the 2.5 kcal/mol threshold window of relative energy were discarded. The selected pairs of conformers were prone to single-point energy computations using the def2-TZVPD basis set with the fine grid tetrahedron cavity and the inclusion of parameter sets with hydrogen bond interaction and the van der Waals dispersion term based on the “D3” method of Grimme et al. [69]. These final computations are performed to preserve consistency with monomer characteristics. The values of Gibbs free energies were determined using COSMOtherm with BP_TZVPD_FINE_21.ctd parametrization. Cluster energies were additionally characterized by the inclusion of corrections accounting for zero-point vibrational energy (RI-DFT BP86 (B88-VWN-P86)/def2-TZVPD level) and electron correlation (RI-MP2/def2-QZVPP level). Moreover, the BSSE was estimated using the DFT-C approach [70], the formulation of which includes atom–atom many-body corrections and which is a parameterized geometry-based method. All energy corrections were calculated for each conformer of each cluster, averaged with weights corresponding to the population fraction estimated using Boltzmann probability. Hence, $\Delta E_{g}^{\mathrm{cor}},$ $\Delta E_{g}^{\mathrm{ZPE}},$ and $\Delta E_{g}^{\mathrm{BSSE}}$ characterize the correction for a given pair including contributions coming from all conformations of a given cluster.

The solute–solvent affinity was represented by the values of the Gibbs free energy corresponding reactions, ΔG_r_, of pair formation X + Y = XY, where X and Y represent either solute or solvent molecules. In the case of X = Y, dapsone dimers are formed, and in the case of X ≠ Y, solute–solvent heteromolecular binary contacts are considered. It is worth mentioning that computations of ΔG_r_ might result either in a concentration-independent measure defined based on activity values, ΔG_r_(a), or concentration-dependent items defined using a mole fraction, ΔG_r_(x). The two are interrelated via the activity coefficient product. For the purpose of machine learning, the latter was used, but for overall affinity characteristics, the former is most suitable. In addition, the values of the Gibbs free energies of a solution of dapsone, dapsone dimers, and dapsone–solvent pairs in a given solvent were extracted from the COSMOtherm output files. These values were used as additional molecular descriptors besides the values of Gibbs free energies of cluster formation. The enthalpic and entropic contributions to the affinities were also included in the pool of molecular descriptors. The whole descriptors dataset is provided in supporting materials (see columns L–T in the “data“ spreadsheet of the File S2).

#### 2.4.3. Machine Learning Protocol

The solubility prediction model was formulated using the in-house Python (ver.3.10, https://www.python.org/) code developed for hyperparameter tuning of 36 regression models. They use a variety of algorithms including linear models, boosting, ensembles, nearest neighbors, neural networks, and also some other types of regressors. The hyperparameter space was explored to find their optimal values using the Optuna study (ver.3.2, https://optuna.org/), a freely available Python packagefor hyperparameter optimization [71]. Model tuning was performed through 5000 minimization trials using the tree-structured Parzen estimator (TPE) as the search algorithm sampler. This computationally efficient model-based optimization algorithm uses a probability density function to model the relationship between hyperparameters and performance metrics. To evaluate the performance of each regression model, a new custom score function was developed that combines multiple metrics to account for both the accuracy and generalizability of the model as defined in the previous work [47], where the mathematical details are provided. The most important aspect of the applied scoring function is the inclusion of the penalties obtained from the learning curve analysis (LCA) of the scikit-learn 1.2.2 library during parameter tuning. Since LCA can be computationally expensive, only two-point computations were performed here by including 50% and 100% of the total data. The LCA evaluations of the final model were performed using 20-point calculations in the range of 50%–100%. The values included in the custom loss correspond to the average MAE values obtained at the largest training set size. Thus, such a custom loss function combines the two types of components and provides information about the model’s accuracy and ability to generalize to new, unseen data. The final performance of all modes was evaluated using the loss values characterizing the test and validation subsets. The ensemble model (EM) was defined by including the subset of regression modes with the lowest values of both criteria, and the final predictions were averaged over selected models.

## 3. Results and Discussion

### 3.1. New Data of Dapsone Solubility

In order to extend the available solubility data for dapsone, a series of measurements were performed with five different organic solvents, namely N-methyl-2-pyrrolidone (NMP), dimethylsulfoxide (DMSO), 4-formylmorpholine (4FM), tetraethylene pentamine (TEPA), and diethylene glycol bis(3-aminopropyl) ether (B3APE), at a temperature range of 298.15 K to 313.15 K. The results of these measurements are summarized in Table 1.

Some interesting observations can be made when analyzing the presented results. At a temperature of 298.15 K, the highest solubility of dapsone was found in the case of DMSO with x_DAP_ = 187.57·10^−4^, and a decreasing solubility trend of DMSO > B3APE > TEPA > NMP > 4FM is present. With increasing temperature, the DAP solubility also increases, although not in an even manner for all studied solvents. The highest solubility increase from 298.15 K to 313.15 K was observed for NMP (of 10.6 times in mole fractions) with the lowest in the case of DMSO (of 2.4 times in mole fractions). This resulted in a change in the overall solubility trend with NMP yielding the highest solubility of dapsone at 313.15 K, amounting to x_DAP_ = 1113.63·10^−4^.

### 3.2. Solubility Prediction Using Consonance Solvents

**Figure 1:**
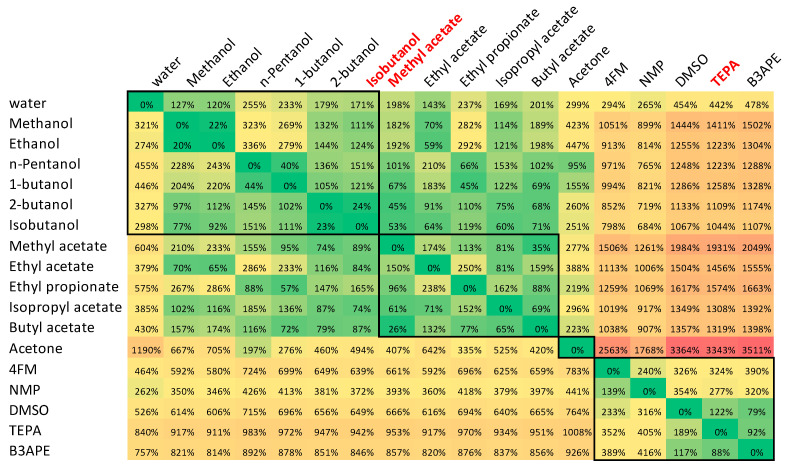
The collection of MAPE (mean absolute percentage error) values for all combinations of solvent–reference solvent solubility calculations. The left column denotes the solvent in which the solubility is calculated, and the first row enumerates the reference solvents. Thus, the diagonal represents solubility calculations in a given solvent with itself as a reference, and, therefore, the MAPE is equal to zero. In red color, the names of the consonant solvents are marked, which minimize the MAPE for the subset of solvents enclosed by a rectangle.

The COSMO-RS framework is often used for theoretical solubility evaluations. However, since the approach is defined for fluids, its application to saturated solid–liquid systems is only possible if information on the melting properties is available. Unfortunately, in many cases, these data are not available due to a lack of reported measurements or, even worse, due to the impossibility of making such measurements. Many solids cannot be studied by using the classical DSC approach due to thermal instabilities or complex phase transitions [72,73,74]. This is exactly the case for dapsone [55,75]. Solid DAP exhibits quite complex equilibria as it can exist in five anhydrous forms [76]. It happens so that polymorph III is predominantly present in commercial products as the most stable in ambient conditions. Unfortunately, this polymorph undergoes a transition to form II below the melting point [55,75]. Therefore, it is not possible to accurately measure the melting temperature of this form, which exists in the equilibrated saturated solutions under conditions of solubility determination. Consequently, the experimental determination of the enthalpy of fusion and the heat capacity is also prohibited due to this circumstance. This inherent limitation can be overcome by estimating T_m_ and H_fus_ using, for example, the group additive approach, as documented by Li et al. [55]. However, this introduces errors in the solubility computations, the significance of which is difficult to estimate. Therefore, for the purpose of validating the ability of COSMO-RS to predict the solubility of DAP, a different strategy was adopted using a method called the consonance solvent approach [64]. This approach is based on the identification of reference solvents for which the solubility is known and using these data to indirectly calculate the thermodynamic properties of the melt. Therefore, for the purpose of this study, all possible combinations of reference solvents were considered to estimate the solubility of dapsone. The results are presented in the form of a heat map, as visualized in Figure 1. The heat map collects the values of the MAPE determined for all possible combinations of solvent–reference solvents. Interestingly, this allows for the grouping of solvents with a similar accuracy of solubility calculations and then for finding the best set of matching solvents. According to chemical intuition, COSMO-RS-derived solubilities are expected to be of similar accuracy for closely related chemical structures of solvents. Indeed, all proton-donating solvents were included in the first subset. Therefore, using alcohol as a reference solvent provided the best estimate of solubility for alcohols. In this case, isobutanol (iBuOH) seems to be the best choice. For DAP solubility estimation in proton-accepting solvents, it is logically better not to use alcohol but rather the solvent belonging to the same subgroup. Here, ethylene propionate (EtPr) seems to be the best choice for this class of solvents. The last subset identified in Figure 1 includes other polar non-protic solvents, and TEPA was identified as the best choice. Thus, the procedure led to the determination of the minimal set of optimal selection of three reference solvents for minimizing the error of the COSMO-RS derived solubility. A closer inspection of the lower left rectangle of the heat map clearly shows a similarity between NMP and 4FM. The remaining three solvents classified in this region, namely DMSO, TEPA, and B3APE, should represent a distinct subset since the values of percentage error have the opposite sign. To keep the number of congruent solvents to a minimum, only three classes are accepted. However, acetone is in a class of its own and can hardly be considered a reasonable reference solvent, and vice versa—no other solvent can serve as a reference for acetone. Therefore, acetone was excluded from the pool of reference solvents, or to put it another way, it can be expected that the class of other ketones might emerge from this analysis. In the end, four reference solvents were defined that resembled the structural diversity of the solvents in the dapsone solubility dataset. However, despite the efforts made to optimize the solubility calculations, the accuracy of the values obtained is still hardly acceptable, which can be directly deduced from the MAPE values provided. Considering that this statistical measure is calculated on the basis of the decadal logarithm of the mole fraction, the overall accuracy of solubility calculations using the COSMO-RS approach is at best qualitatively related to the measured values. This was the direct impulse for the development of nonlinear models and the formulation of a quantitative tool. To further illustrate this need, the values computed using the optimized set of consonant solvents are confronted with the predictions made by the ensemble of regressor models in the next section.

### 3.3. Solute–Solvent Intermolecular Interactions

**Figure 2:**
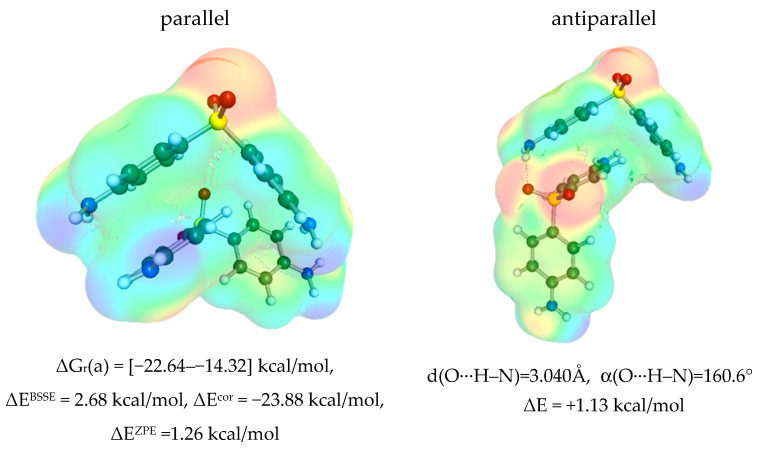
The self-association properties of DAP. The electron charge density profiles of the two most probable conformations in all studied solutions were supplemented with energetic contributions of self-association.

The selection of the molecular descriptors is a crucial step in the machine learning protocol. There are many possible parameters, which are used by different authors [77,78,79,80,81], for machine learning. However, the actual selection is limited by the fact that the temperature dependence has to be explicitly included for an adequate description of the pool of experimental dapsone solubility. Fortunately, there are many valuable molecular descriptors that could be derived using the COSMO-RS approach, such as calculated values of chemical potential, activities, or different energetic contributions. It has already been documented that they can serve as quite reasonable quantities for training purposes [47]. Nevertheless, an alternative set of molecular properties was tested here. Solute–solute and solute–solvent affinities were determined as described in the ‘Materials and Methods’ section. Thus, the most likely homo- and heteromolecular pairs were screened, for which the values of the Gibbs free energies of the synthesis reaction from monomers were determined. The first step was to analyze the self-association of two DAP molecules. Although dapsone can be considered a molecule with potential proton acceptor and proton donor centers, the self-association of two DAP molecules adopts a stacking conformation in all solvents analyzed rather than stabilization by hydrogen bonding. Conformational analysis identified two types of stacking complexes in which either a parallel or antiparallel orientation of two DAP molecules occurs, as documented in Figure 2.

Despite the fact that in the antiparallel orientation, the hydrogen bond is formed between the sulfanyl group of one molecule and the amino side group of the other, this structure is less stable by about 1.1 kcal/mol. This suggests a marginal contribution to the overall thermodynamic properties of DAP. The necessary distortion of the amino group is responsible for such a destabilizing effect. Moreover, the shape of the HB is not expected to be strong, as suggested by the low value of the O···H–N angle.

It is worth mentioning that the stacking conformation is stable in all saturated systems considered and is the most favorable intermolecular complex among all solute–solvent contacts considered here. A fairly linear trend between solubility and DAP stacking affinity is observed, suggesting that this could be a promising descriptor for solubility prediction via machine learning. It is also worth mentioning that the dispersion forces are very strong in the case of DAP stacking, as documented by the values of the electron correlation contribution to the total energy. The contributions from ZPE and BSSE are of opposite signs but only slightly reduce the DAP self-affinity. It is also worth noting that there is a modest correlation of the ΔG_r_ of DAP self-association estimated in different solvents with solubility (R^2^ = 0.7), which is a good prognosis for using these values as molecular descriptors in the nonlinear model training. As expected, the self-affinity of DAP is highest in the case of water, which has the lowest solubility. In contrast, the highest solubility observed in DMSO is associated with the weakest self-affinity of dapsone. Since the stacking of dapsone is similar to the interactions in the crystal, it is reasonable to expect that the promotion of self-association will lead to clustering, which will make it difficult to disperse in the bulk solvents, eventually leading to precipitation. Therefore, the coincidence of solute and solvent in this hydrophobic region seems to be the most important solubility factor. To further explore this suggestion, the heteromolecular complexes potentially present in the solvents analyzed were studied.

**Figure 3:**
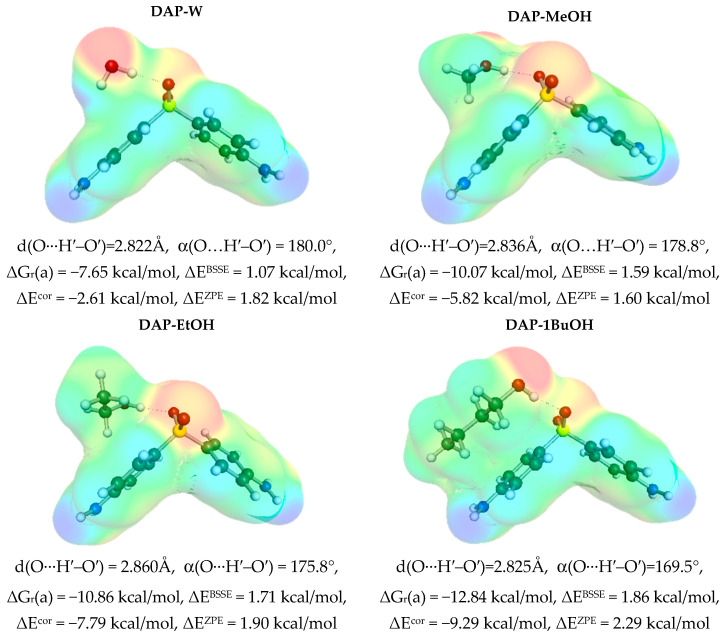

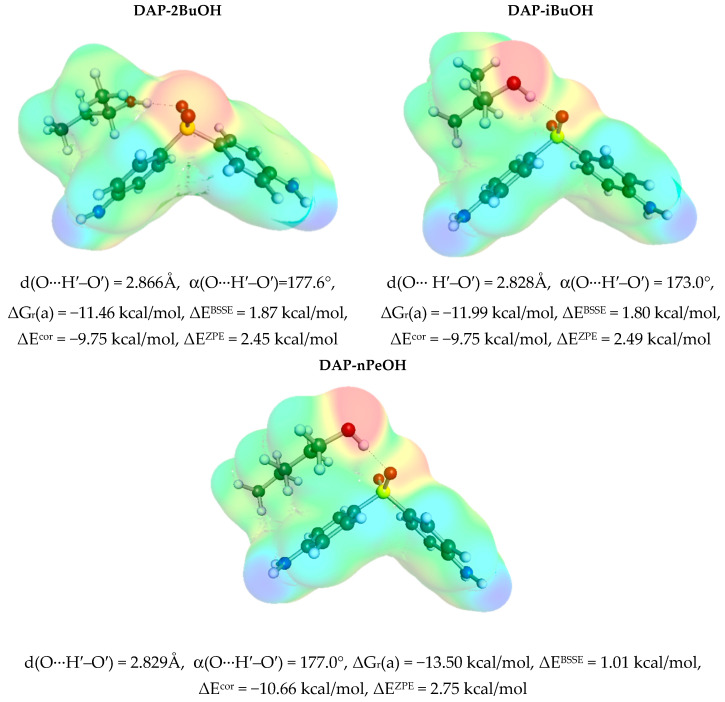
The characteristics of solute–solvent contacts of DAP in proton-donating solvents like water and alcohols. Prime denotes atoms on HBD counter partner in hydrogen bonding.

Dapsone has weak hydrogen-donating and -accepting properties, allowing interactions with a variety of solvent molecules. The former interactions are provided by the peripheral amine groups, while the latter are provided by the two oxygen atoms on the sulfonyl group. In addition, as mentioned in the context of self-association, DAP has wide delocalized electron clouds that allow for non-specific interactions. This is granted by the pocket-like region formed by two phenyl groups, which form a nonpolar interior attractive for all molecular fractions of a nonpolar nature. Therefore, it is expected that dapsone will be stabilized by different intermolecular interactions with solvents depending on their nature. In fact, upon closer inspection of the structure of the most probable pairs, it is possible to distinguish three classes of systems. In Figure 3, the examples of pairs are presented in which DAP acts as a proton acceptor via the sulfanyl group. These affinities vary from −7.7 kcal/mol in the case of water up to −13.5 kcal/mol for nPeOH (n-pentanol). In all cases, hydrogen bonds are formed that could be classified as strong based on their geometric parameters. The presented structures show that the region of the DAP molecule active in self-association is not affected by all these solute–solvent interactions, suggesting that higher-order trimers or clusters are quite likely. Unfortunately, the size of these contacts prohibits the advanced calculations used in this project. However, this speculation might suggest that the solubility in these proton-donating solvents is expected to be at most modest due to possible dapsone self-association driven by non-specific interactions.

**Figure 4:**
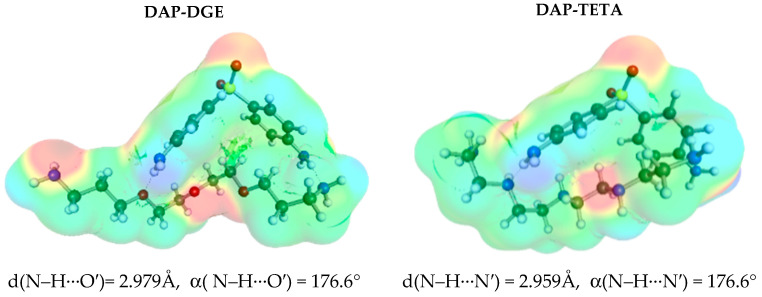

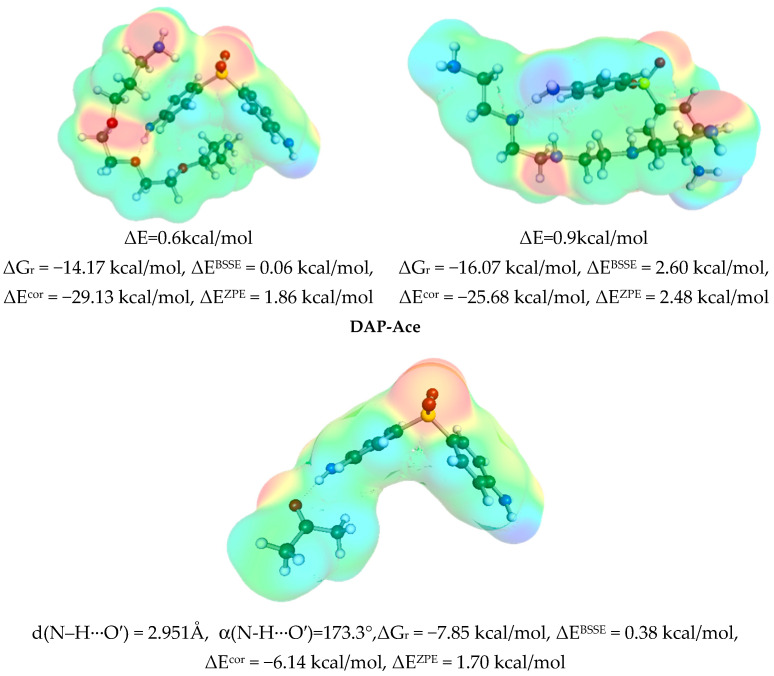
The solute–solvent contact characteristics of DAP in polar proton-accepting solvents such as acetone and esters.

In contrast, the second class of solvents collected in Figure 4 includes contacts in which DAP acts as a proton donor. This class includes acetone, DGE (diethylene glycol bis(3-aminopropyl) ether), and TETA (tetraethylene pentamine). It is worth noting that the molecules of the latter two solvents, due to their chain size, are also attracted to the apolar pocket of dapsone. Therefore, it is expected that there is a coincidence between self-association and solvents, which could be used as a rationale for the very high solubility of DAP in these solvents. The molecular mechanism is quite clear. By blocking the nonpolar region, DAP is less prone to self-associate and form larger clusters, promoting dispersion rather than precipitation. Even a small molecule of acetone makes DAP dimerization difficult by forming very strong hydrogen bonds and partially occupying the nonpolar pocket.

**Figure 5:**
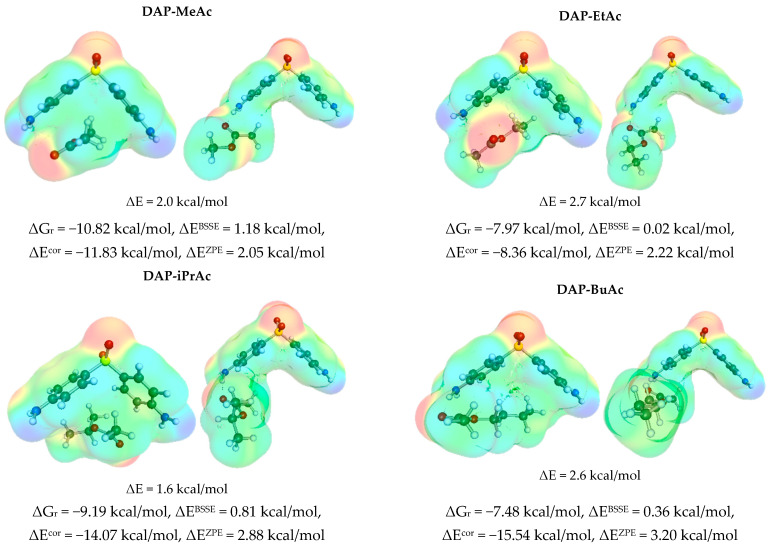

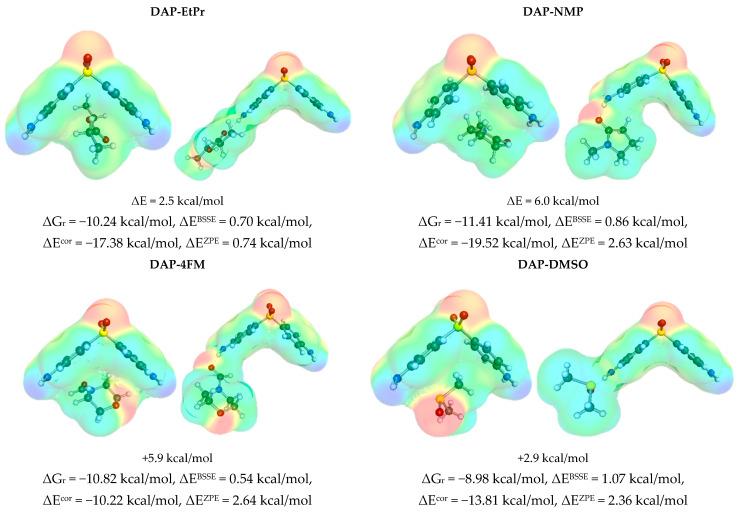
The characteristics of solute–solvent contacts of DAP in polar non-proton-containing solvents in which dapsone interacts mainly via non-HB interactions.

The third class represents solvents that interact with DAP via non-HB interactions. These compounds are typical polar aprotic solvents such as those used in this study, namely NMP, 4FM, and DMSO, as well as others taken from the literature, namely MeAc (methyl acetate), EtAc (ethyl acetate), iPrAc (isopropyl acetate), BuAc (butyl acetate), and EtPr (ethyl propionate). Interestingly, the ΔGr values calculated for the complexes formed by DAP and these solvents (Figure 5) generally do not differ significantly from the previously discussed classes.

### 3.4. Ensemble Model for Solubility Prediction

Although the consonance solvent approach allows solubility calculations for dapsone despite the lack of fusion thermodynamic properties, the accuracy of this approach is far from acceptable. Therefore, machine learning techniques were used to develop a more reliable solubility prediction model. The methodology used in this study is similar to our previous work [47], although a new set of molecular descriptors was used. As discussed above, the solubility of dapsone can be rationalized in terms of intermolecular interactions in the saturated solutions. Consequently, the machine learning protocol included affinities as well as entropic and enthalpic contributions for regressor training. All molecular descriptors, predicted solubilities, and model details are provided in the supporting materials (see the File S2).

**Figure 6:**
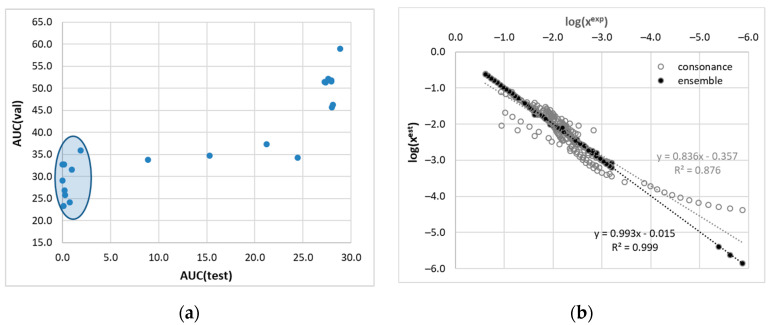
Results of machine learning analysis of dapsone solubility in pure solvents and binary mixtures. (**a**) AUC (area under the curve) derived from the LCA using 5-fold cross-validation. (**b**) Correlation between calculated and measured solubility data. The black circles indicate the predictions of the ensemble, while the open circles document the results of consonance solvation using four reference solvents (MeAc, iBuOH, TETA, and Ace).

**Figure 7:**
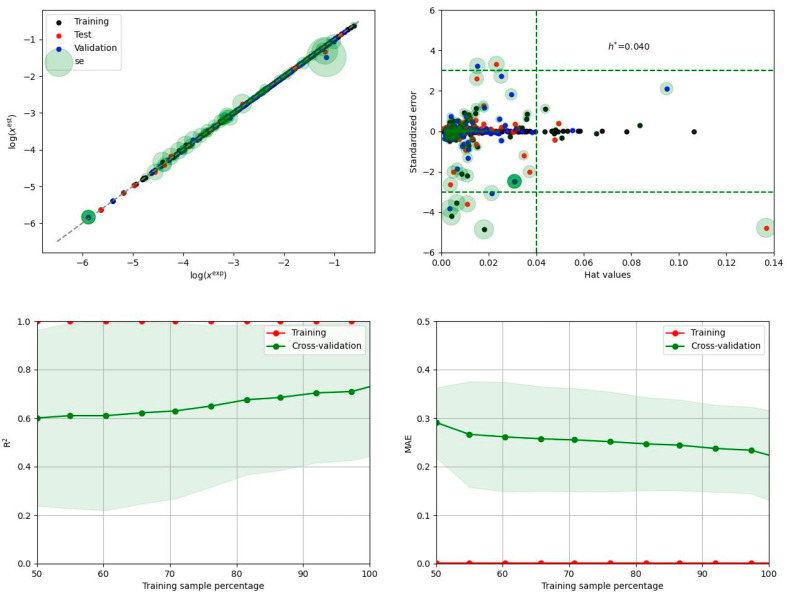
Graphical representation of the performance of the NuSVR regressor, which was found to be best suited for dapsone solubility calculations based on molecular descriptors derived from solute–solvent intermolecular interactions. The top panel shows a correlation between calculated and measured solubility data and the applicability domain plot. Bottom panel illustrates the results of learning curve analysis (LCA) and area under the curve (AUC) determination.

In particular, the focus of the regressor model tuning effort went beyond minimizing the deviation between computed and experimental data. The primary goal was to improve the predictive power of the developed model. To accomplish this, a custom loss function was employed that included a penalty estimated via learning curve analysis. While this approach may slightly compromise the fitting accuracy, it significantly improves the predictive potential of the model. From the 36 tunable models included in our Python code, a final set of nine regressor models was selected for ensemble construction. The inclusion criterion was derived from the learning curve analysis using five-fold cross-validation. Figure 6a illustrates the relationship between the area under the curve (AUC) for the validation and training subsets, clearly showing a distinct cluster of nine regressors. The order in which the models are listed reflects the increasing value of the AUC derived from the root mean square (RMS) plot obtained by systematically increasing the data portion from 50% to 100% using learning curve analysis. The ensemble of regressors developed in this study includes a diverse set of machine learning algorithms, each with unique characteristics. The aggregate consists of NuSVR, SVR, MLPRegressor, CatBoostRegressor, RandomForestRegressor, BaggingRegressor, HistGradientBoostingRegressor, KNeighborsRegressor, and ExtraTreeRegressor models. These models offer various learning strategies and approaches. Support vector machines (SVMs), such as NuSVR and SVR, excel at finding hyperplanes that separate data or predict continuous values, providing flexibility and adaptability for solving different regression problems. Random forests and extra trees employ ensemble learning techniques based on decision trees, while gradient boosting methods (e.g., HistGradientBoostingRegressor or CatBoostRegressor) sequentially build ensembles of weak learners. K-nearest neighbors considers data point proximity for predictions. The CatBoostRegressor is a powerful machine learning algorithm that is well suited for solubility modeling due to its ability to effectively detect complex relationships between molecular descriptors and solubility. The MLPRegressor takes advantage of the artificial neural networks, which provide a flexible infrastructure for capturing nonlinear relationships by allowing the specification of the number of hidden layers and their respective sizes. This flexibility enables the model to effectively represent complex patterns and dependencies present in the solubility data. To optimize the performance of all of these models, an extensive tuning process was performed using an Optuna study. This involved optimizing the hyperparameters of the models to identify the best-performing configurations. As a result, the ensemble exhibits diversity in terms of the hyperparameter settings chosen across the different models. By integrating these different models into an ensemble, one can leverage their individual strengths while mitigating their weaknesses. The use of an ensemble approach improves predictive performance by aggregating the predictions of multiple models, allowing a wider range of patterns to be captured and reducing the impact of individual model biases. In the case of the dapsone solubility model, the ensemble is constructed simply by averaging the predictions provided by each regressor without optimizing their weight. It is important to note that when predicting solubility expressed in terms of the decadal logarithm of the mole fraction, a formal constraint is imposed on the solution. Therefore, only negative predicted values are considered for averaging. Fortunately, the ensemble components consistently met this requirement. The developed ensemble of regressors provides a robust framework that leverages the strengths of machine learning algorithms, resulting in highly accurate predictions, as evidenced by the results shown in Figure 6b. The performance of the aggregated regressors exceeds the accuracy of solutions obtained using the consonance solvent approach. To illustrate the individual performance of the regressors, the compilation is offered in the supporting materials (see Section S2 in File S1). For illustrative purposes, the results of the best-performing model are documented in Figure 7.

The power of even a single regressor to accurately predict dapsone solubility is evident. This is demonstrated not only by the near-perfect agreement between calculated and experimental values but also by the smooth trend observed in the learning curve analysis (LCA) plot. As the percentage of data included in the LCA increases, there is a systematic and gradual improvement in the resulting R2 and MEA, indicating the robustness and stability of the model predictions.

## 4. Conclusions

The dissolution of dapsone, as an important aspect from both practical and theoretical points of view, can be rationalized based on its ability to form intermolecular complexes in saturated solutions. The structure of DAP suggests that this solute can interact with solvent molecules in a variety of ways. Solute–solvent contacts can be stabilized by proton acceptor and proton donor centers present in the DAP molecule, suggesting the promotion of dissolution in solvents, the structures of which provide a counterpart for hydrogen bonding. Moreover, broad delocalized electron clouds of aromatic rings are favorable regions for interactions with nonpolar solvents or those containing nonpolar fragments. It is therefore quite surprising that DAP is not too soluble in most organic solvents and their mixtures. The clue to this apparent paradox may be provided by the study of the self-association of two DAP molecules. The results presented suggest that the homomolecular contacts adopt a stacking conformation in all pure solvents analyzed and that this type of cluster is the most stable among all other types of binary complexes. The strong tendency to self-associate is due to nonpolar interactions similar to the close contacts observed in the solid state. There is a very high contribution from electron correlation, which further enhances the stability of DAP dimers. Thus, the high self-affinity of dapsone is responsible for its aggregation, which eventually leads to precipitation regardless of the solvent. However, solvents that can disrupt dapsone stacking by at least partially blocking the apolar regions act as better dispersants and allow higher concentrations in saturated conditions. It is important to note that the elucidation of this mechanism may have been due to the fact that the solvent space was significantly expanded by providing new solubility data in such solvents. Without such new experimental data, the mechanism underlying the dissolution of dapsone would not be clear, as there are no accurate models to predict solubility values in solvents in which DAP has not been measured. This has been documented by COSMO-RS calculations, whose predictions have at most qualitative accuracy. Furthermore, other existing models are not helpful in this respect. For example, recently, an interesting approach using the 2D structural data (SMILES code) of the solvent and solute for the prediction of the solubility at molar concentrations has appeared [45,82]. In general, the concept of avoiding quantum chemical computation is very attractive due to its efficiency. Unfortunately, there are unsatisfactory high deviations (>100%) when comparing the online prediction results with new experimental data presented in Table 1.

Therefore, the ensemble of nonlinear models was defined based on nine regressors. The effectiveness of the Optuna study allows for hyperparameter tuning and tailoring of the ensemble of models to solve a particular problem. It is interesting to note that the values of intermolecular parameters such as contact affinities and solvability carried sufficient information to formulate a very accurate model of dapsone solubility in neat solvents and their binary mixtures. Despite the very high accuracy of the provided model, it has some disadvantages related to the set of molecular descriptors, since the characteristics of intermolecular interactions are computationally expensive. However, the value of the obtained information about the structure, affinities, and molecular descriptors is worth the effort. In principle, the developed model allows screening in any type of solvent if only the contact characteristics are provided.

## Figures and Tables

**Table 1:** The solubility of dapsone in neat organic solvents at various temperatures expressed as molar concentration s_DAP_ and mole fraction (x_DAP_, ·10^4^). Standard deviation values are given in parentheses.

T (K)	s_DAP_ (mol/dm^3^)					x_DAP_∙10^4^				
	4FM	DMSO	TEPA	NMP	B3APE	4FM	DMSO	TEPA	NMP	B3APE
298.15	0.042 (±0.000)	0.256 (±0.009)	0.056 (±0.001)	0.108 (±0.003)	0.068 (±0.001)	43.11 (±0.25)	187.57 (±7.02)	105.69 (±2.23)	105.13 (±3.20)	147.51 (±1.43)
303.15	0.074 (±0.004)	0.344 (±0.002)	0.079 (±0.002)	0.243 (±0.005)	0.109 (±0.011)	73.90 (±4.18)	251.58 (±1.54)	149.90 (±4.00)	236.59 (±5.16)	231.14 (±22.69)
308.15	0.126 (±0.001)	0.462 (±0.012)	0.104 (±0.000)	0.525 (±0.009)	0.142 (±0.002)	125.57 (±0.89)	342.88 (±9.24)	196.54 (±0.47)	524.74 (±10.19)	297.60 (±4.48)
313.15	0.191 (±0.001)	0.598 (±0.011)	0.141 (±0.002)	1.040 (±0.118)	0.191 (±0.003)	191.48 (±0.55)	444.27 (±9.20)	262.29 (±4.27)	1113.63 (±17.53)	397.51 (±6.06)

## Data Availability

All data supporting the reported results are available on request from the corresponding author.

## Supplementary Materials

The following are available online at https://www.mdpi.com/article/10.3390/ma16186336/s1, (a) in File S1, dapsone solubility data curation and normalization and regressor model performance are provided; (b) in the File S2, all experimental and computed solubility data, molecular descriptors, and optimized parameters of nine regressors are collected.
