# Supplementary material for: Intermolecular Interactions as a Measure of Dapsone Solubility in Neat Solvents and Binary Solvent Mixtures
Source: Materials (Basel). 2023 Sep 21;16(18):6336. doi: 10.3390/ma16186336 (PMC10532775; doi:10.3390/ma16186336)
Supplement: Supplementary file 1 [file materials-16-06336-s001.zip › File S1.pdf]

Supporting materials

Intermolecular interactions as a measure of dapsone solubility in neat solvents and binary solvents mixtures

Piotr Cysewski*, Maciej Przybyłek and Tomasz Jeliński

S1. Dapsone solubility data curation and normalization 3

Table S1. The collection of the fitted values of three parameters van't Hoff equation characterizing dapsone solubility in neat solvents. 3

Figure S1. Illustration of the fitting of dapsone solubility using van't Hoff equation for obtaining the consensus dataset for polar-protic solvents, namely Methanol (MeOH), Ethanol (EtOH), 1-butanol (nBuOH), 2-butanol (2BuOH), Isobutanol (iBuOH), n-Pentanol (nPeOH) and water (W). The symbols [1] and [2] used in the legend stands for [Li, W.; Ma, Y.; Yang, Y.; Xu, S.; Shi, P.; Wu, S. Solubility measurement, correlation and mixing thermodynamics properties of dapsone in twelve mono solvents. J. Mol. Liq. 2019, 280, 175–181] and [Li, H.; Xie, Y.; Xue, Y.; Zhu, peizhi; Zhao, H. Comprehensive insight into solubility, dissolution properties and solvation behaviour of dapsone in co-solvent solutions. J. Mol. Liq. 2021, 341, 117403], respectively. 4

Figure S2. Illustration of the fitting of dapsone solubility using van't Hoff equation for obtaining the consensus dataset for polar aprotic-solvents, namely Methyl acetate (MeAc), Ethyl propionate (EtPr), Butyl acetate (BuAc), Ethyl acetate (EtAc), Isopropyl acetate (iPrAc), Acetone (Ace). The symbols [1] and [2] used in the legend stands for [Li, W.; Ma, Y.; Yang, Y.; Xu, S.; Shi, P.; Wu, S. Solubility measurement, correlation and mixing thermodynamics properties of dapsone in twelve mono solvents. J. Mol. Liq. 2019, 280, 175–181] and [Li, H.; Xie, Y.; Xue, Y.; Zhu, peizhi; Zhao, H. Comprehensive insight into solubility, dissolution properties and solvation behaviour of dapsone in co-solvent solutions. J. Mol. Liq. 2021, 341, 117403], respectively. 5

Figure S3. Illustration of the fitting of dapsone solubility using van't Hoff equation for obtaining the consensus dataset for alcohols and water. The symbols [1] and [2] used in the legend stands for [Li, W.; Ma, Y.; Yang, Y.; Xu, S.; Shi, P.; Wu, S. Solubility measurement, correlation and mixing thermodynamics properties of dapsone in twelve mono solvents. J. Mol. Liq. 2019, 280, 175–181] and [Li, H.; Xie, Y.; Xue, Y.; Zhu, peizhi; Zhao, H. Comprehensive insight into solubility, dissolution properties and solvation behaviour of dapsone in co-solvent solutions. J. Mol. Liq. 2021, 341, 117403], respectively. 6

S2. Regressors models 7

Figure S4. The graphical representation of the performance of SVR regressor. The meaning of the panels is the same as in Figure 7 in the main text, namely top panel provides a correlation of computed and measured solubility data and the applicability domain plot; bottom panel illustrates the results of LCA (Learnig Curve Analysis) and AUC (area under curve) determination. 7

Figure S5. The graphical representation of the performance of MLPRegressor. The meaning of the panels is the same as in Figure S2.1. 7

Figure S6. The graphical representation of the performance of CatBoostRegressor. The meaning of the panels is the same as in Figure S2.1. 8

Figure S7. The graphical representation of the performance of RandomForestRegressor. The meaning of the panels is the same as in Figure S2.1 8

Figure S8. The graphical representation of the performance of BaggingRegressor. The meaning of the panels is the same as in Figure S2.1. 9

Figure S9. The graphical representation of the performance of HistGradientBoostingRegressor. The meaning of the panels is the same as in Figure S2.1.. 9

Figure S10. The graphical representation of the performance of KNeighborsRegressor. The meaning of the panels is the same as in Figure S2.1.. 10

Figure S11. The graphical representation of the performance of ExtraTreeRegressor. The meaning of the panels is the same as in Figure S2.1.. 10

S1. Dapsone solubility data curation and normalization

Table S1. The collection of the fitted values of three parameters van't Hoff equation characterizing dapsone solubility in neat solvents.

$$ln(x_{DAP}^{consensus}) = A + B\left(\frac{1000}{T}\right) + C\left(\frac{1000}{T}\right)^2$$

solvent	A	B	C	RMSD*·10³	MAPE*
Methanol	10.790	-6.777	0.647	39.80	0.76
1-butanol	3.237	-2.991	0.056	35.63	0.46
n-Pentanol	-7.515	3.897	-1.072	25.90	0.32
Methyl acetate	13.078	-9.177	1.242	7.98	0.18
Ethyl propionate	2.968	-4.024	0.497	11.63	0.21
Butyl acetate	1.735	-3.444	0.380	15.25	0.20
Ethanol	11.270	-7.765	0.841	28.19	0.42
2-butanol	-0.370	-0.196	-0.482	17.24	0.21
Isobutanol	34.763	-21.848	2.817	30.76	0.40
Ethyl acetate	0.600	-2.422	0.269	9.26	0.17
Isopropyl acetate	6.851	-6.760	0.932	14.61	0.17
Acetone	15.660	-8.261	0.858	25.30	0.94
water	-10.544	7.514	-2.321	15.56	0.12
4FM	-6.790	10.654	-3.055	16.76	0.32
DMSO	0.426	3.002	-1.285	7.58	0.19
Tetraethylene pentamine	0.052	3.086	-1.327	12.46	0.25
1-Methyl-2-pyrrolidone	39.162	-11.254	-0.530	3.42	0.10
Diethylene Glycol Bis(3-aminopropyl) Ether	-80.050	52.715	-8.973	26.97	0.60

*MAPE (mean absolute percentage error). RMSD (root mean square deviation)

DAP-MeOH

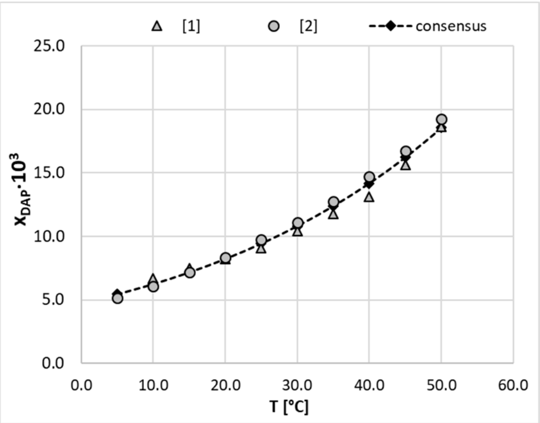

DAP-EtOH

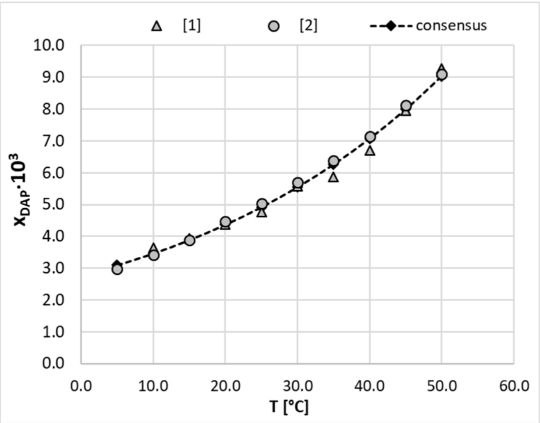

DAP-nBuOH

DAP-2BuOH

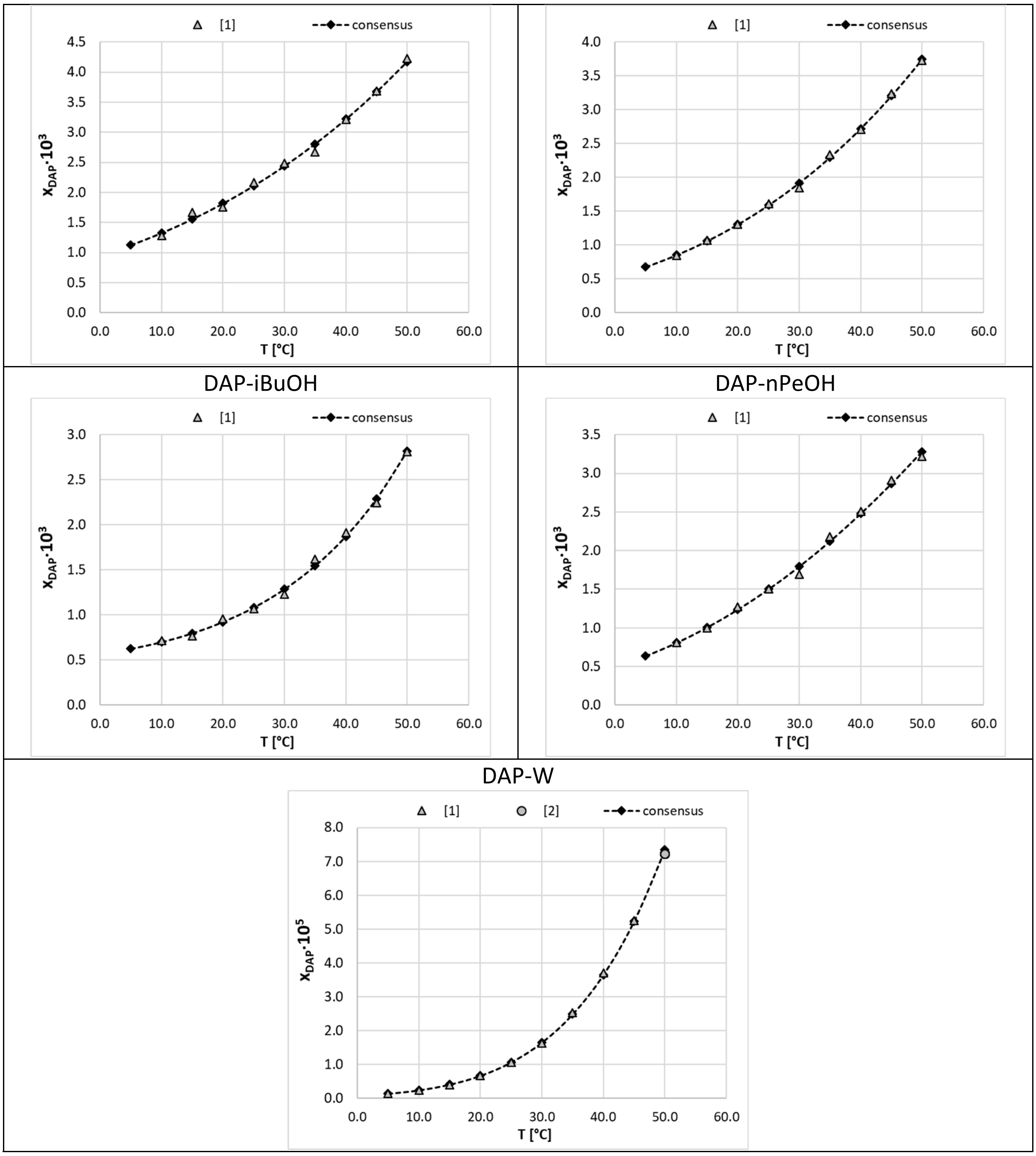

Figure S1. Illustration of the fitting of dapsone solubility using van't Hoff equation for obtaining the consensus dataset for polar-protic solvents, namely Methanol (MeOH), Ethanol (EtOH), 1-butanol (nBuOH), 2-butanol (2BuOH), Isobutanol (iBuOH), n-Pentanol (nPeOH) and water (W). The symbols [1] and [2] used in the legend stands for [Li, W.; Ma, Y.; Yang, Y.; Xu, S.; Shi, P.; Wu, S. Solubility measurement, correlation and mixing thermodynamics properties of dapsone in twelve mono solvents. J. Mol. Liq. 2019, 280, 175–181] and [Li, H.; Xie, Y.; Xue, Y.; Zhu, peizhi; Zhao, H. Comprehensive insight into solubility, dissolution properties and solvation behaviour of dapsone in co-solvent solutions. J. Mol. Liq. 2021, 341, 117403], respectively.

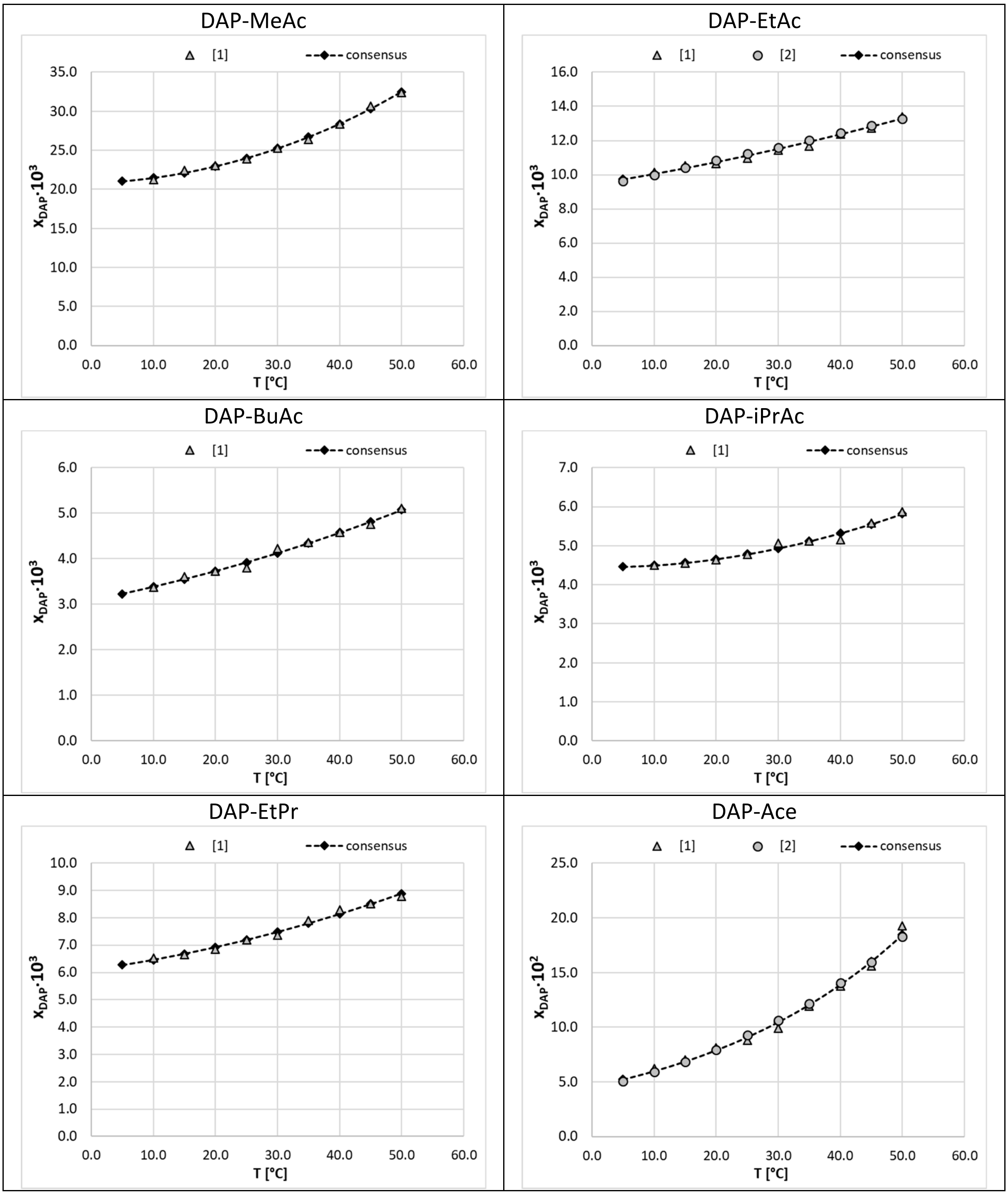

Figure S2. Illustration of the fitting of dapsone solubility using van't Hoff equation for obtaining the consensus dataset for polar aprotic-solvents, namely Methyl acetate (MeAc), Ethyl propionate (EtPr), Butyl acetate (BuAc), Ethyl acetate (EtAc), Isopropyl acetate (iPrAc), Acetone (Ace). The symbols [1] and [2] used in the legend stands for [Li, W.; Ma, Y.; Yang, Y.; Xu, S.; Shi, P.; Wu, S. Solubility measurement, correlation and mixing thermodynamics properties of dapsone in twelve mono solvents. J. Mol. Liq. 2019, 280, 175–181] and [Li, H.; Xie, Y.; Xue, Y.; Zhu, peizhi; Zhao, H. Comprehensive insight into solubility, dissolution properties and solvation behaviour of dapsone in co-solvent solutions. J. Mol. Liq. 2021, 341, 117403], respectively.

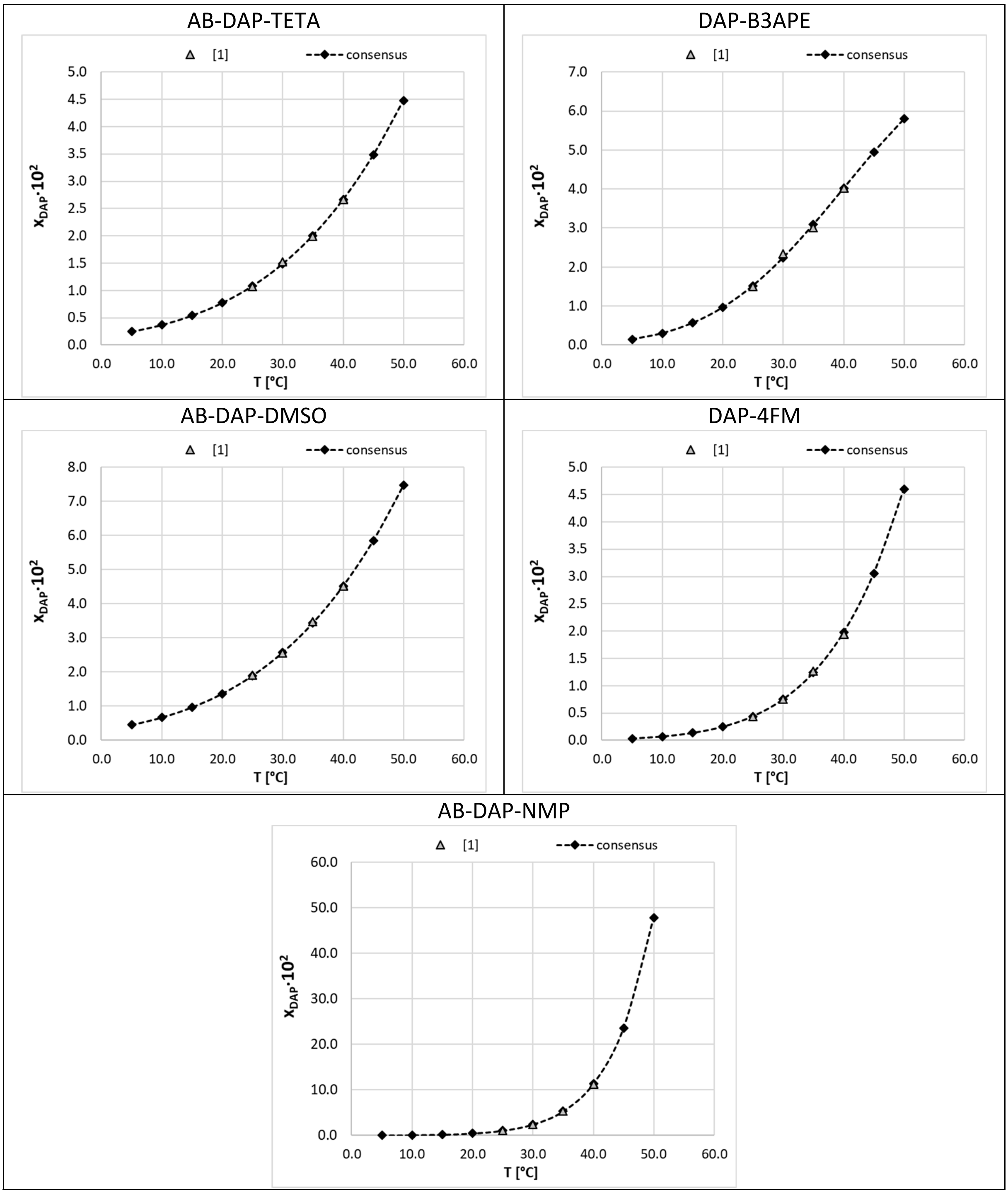

Figure S3. Illustration of the fitting of dapsone solubility using van't Hoff equation for obtaining the consensus dataset for alcohols and water. The symbols [1] and [2] used in the legend stands for [Li, W.; Ma, Y.; Yang, Y.; Xu, S.; Shi, P.; Wu, S. Solubility measurement, correlation and mixing thermodynamics properties of dapsone in twelve mono solvents. J. Mol. Liq. 2019, 280, 175–181] and [Li, H.; Xie, Y.; Xue, Y.; Zhu, Peizhi; Zhao, H. Comprehensive insight into solubility, dissolution properties and solvation behavior of dapsone in co-solvent solutions. J. Mol. Liq. 2021, 341, 117403], respectively.

S2. Regressors models

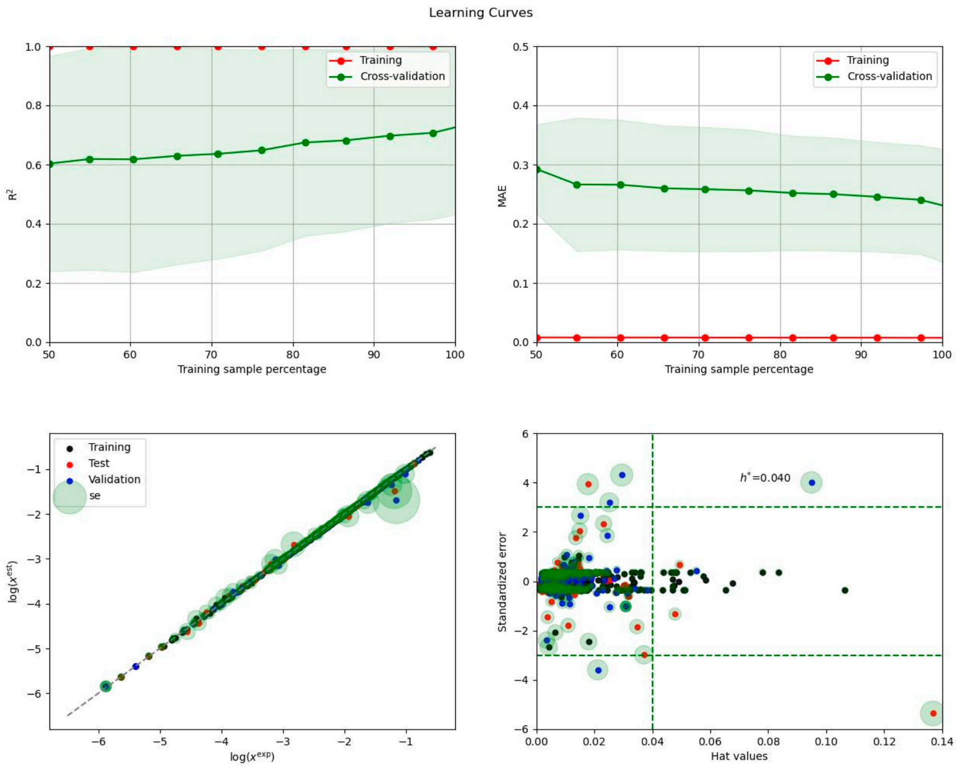

Figure S4. The graphical representation of the performance of SVR regressor. The meaning of the panels is the same as in Figure 7 in the main text, namely top panel provides a correlation of computed and measured solubility data and the applicability domain plot; bottom panel illustrates the results of LCA (Learnig Curve Analysis) and AUC (area under curve) determination.

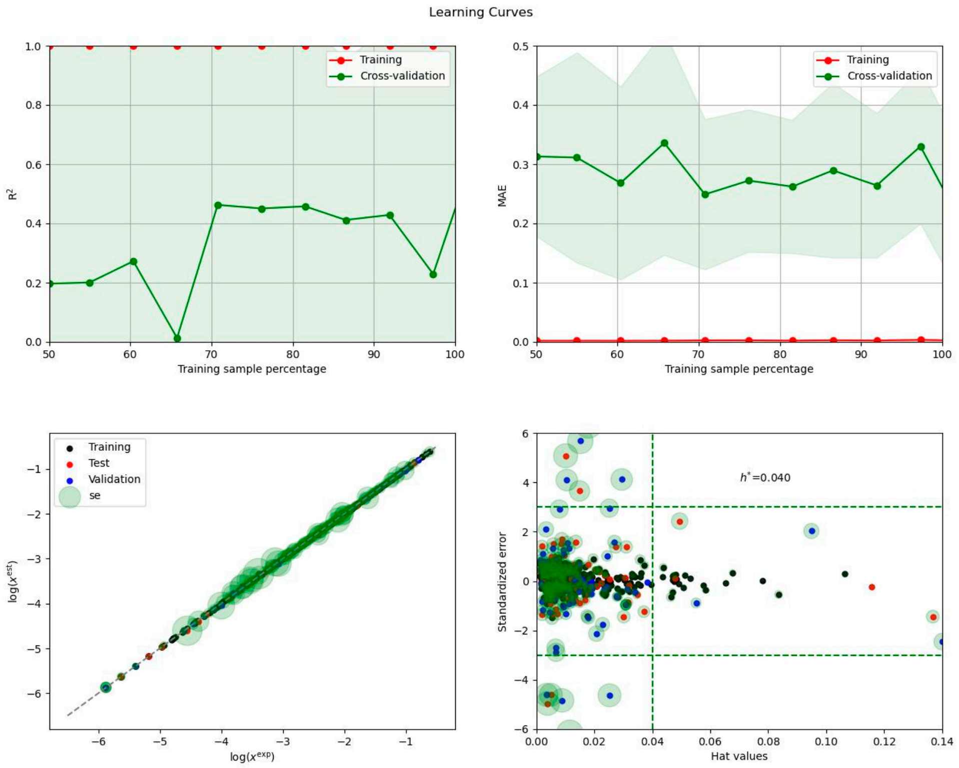

Figure S5. The graphical representation of the performance of MLPRegressor. The meaning of the panels is the same as in Figure S2.1.

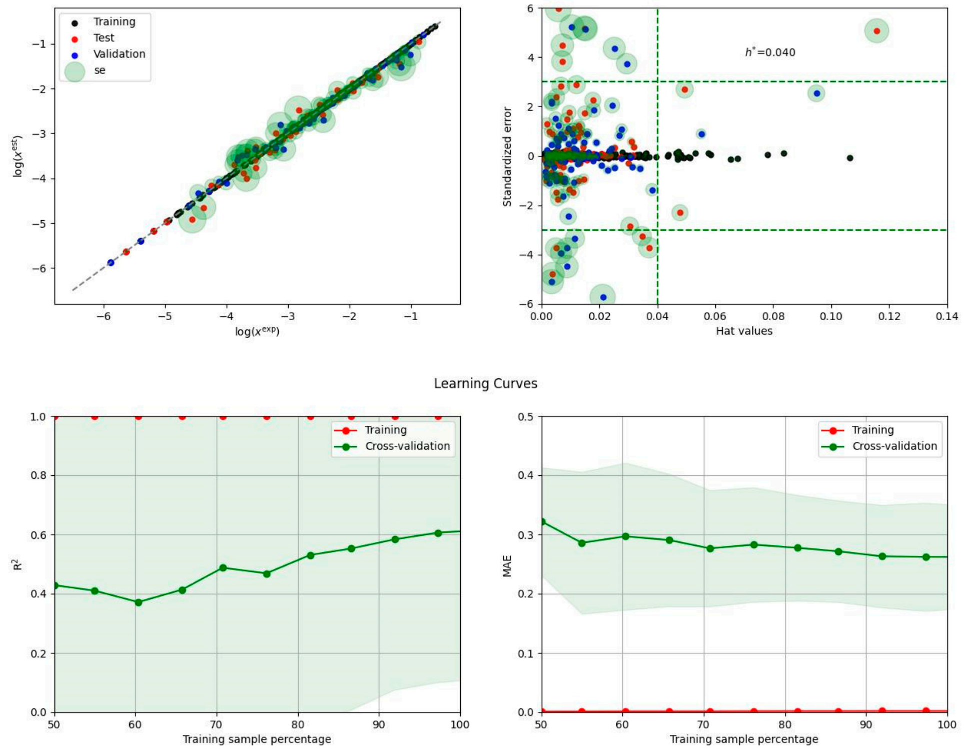

Figure S6. The graphical representation of the performance of CatBoostRegressor. The meaning of the panels is the same as in Figure S2.1.

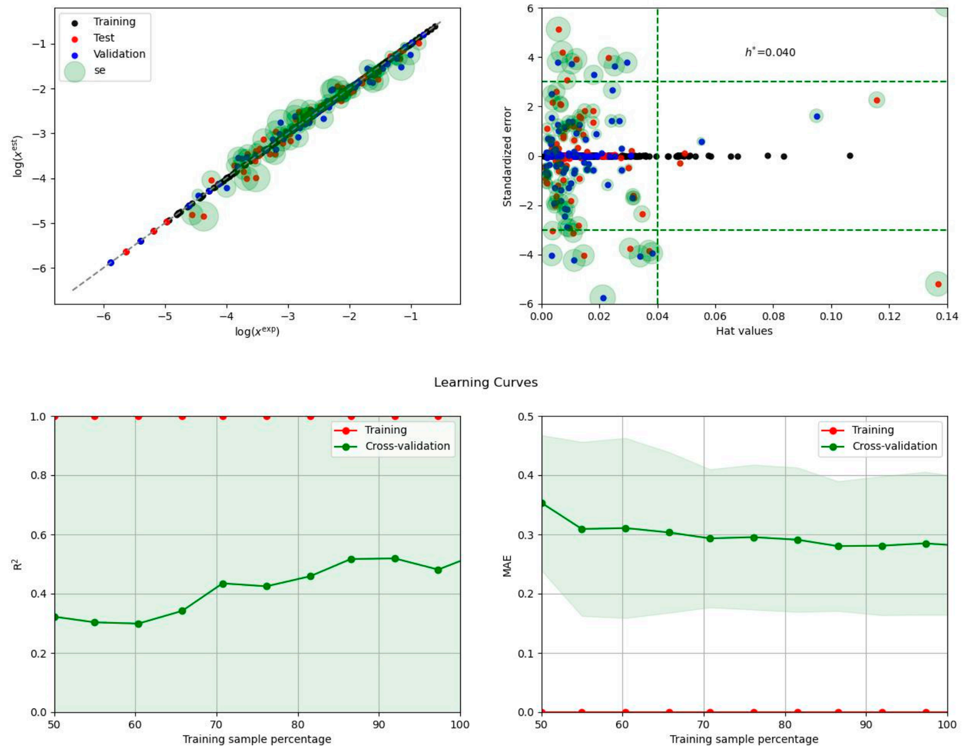

Figure S7. The graphical representation of the performance of RandomForestRegressor. The meaning of the panels is the same as in Figure S2.1.

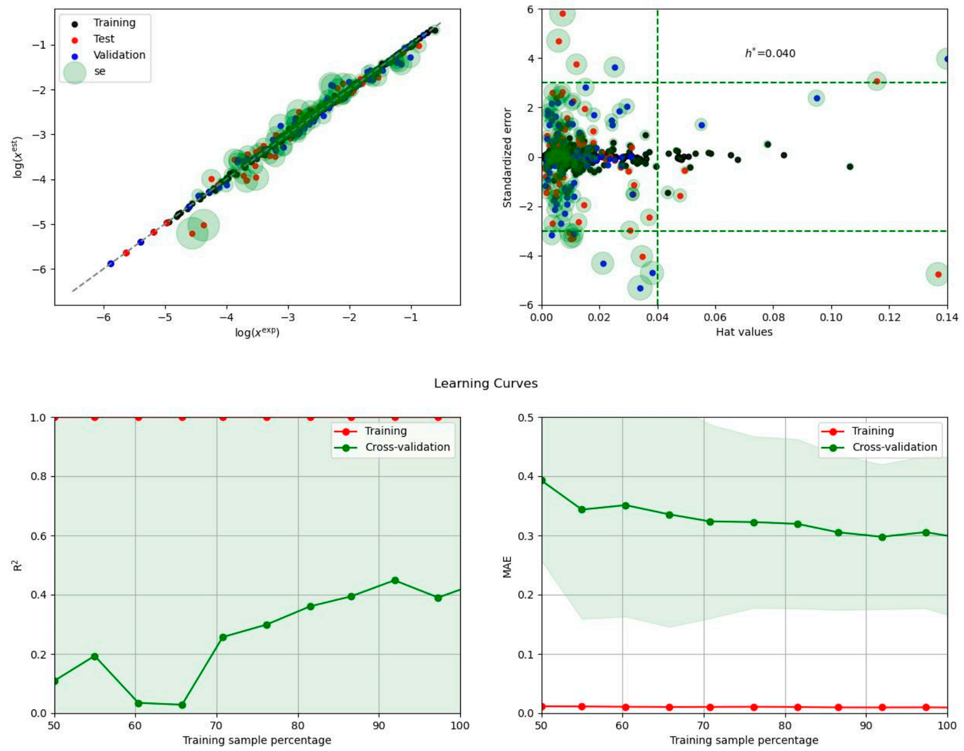

Figure S8 The graphical representation of the performance of BaggingRegressor. The meaning of the panels is the same as in Figure S2.1.

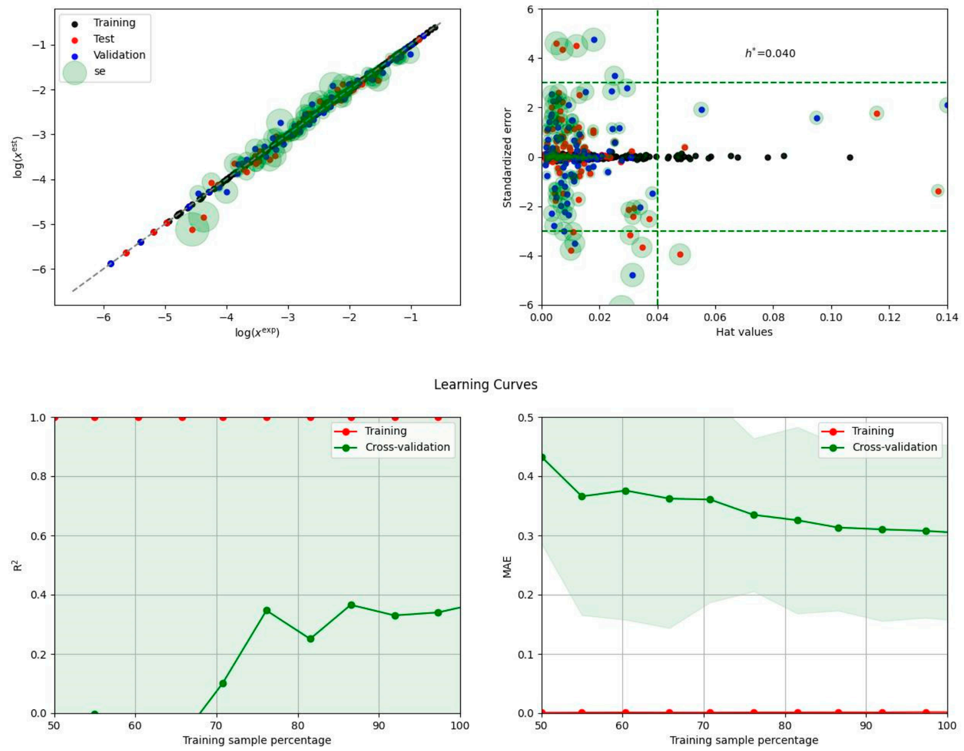

Figure S9. The graphical representation of the performance of HistGradientBoostingRegressor. The meaning of the panels is the same as in Figure S2.1.

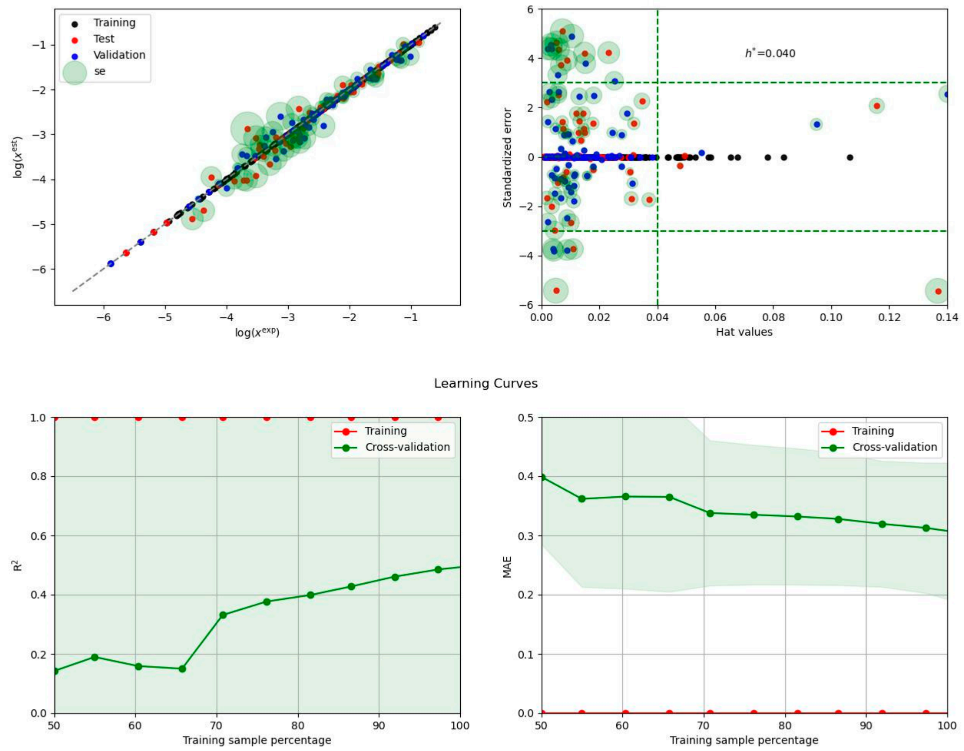

Figure S10. The graphical representation of the performance of KNeighborsRegressor. The meaning of the panels is the same as in Figure S2.1.

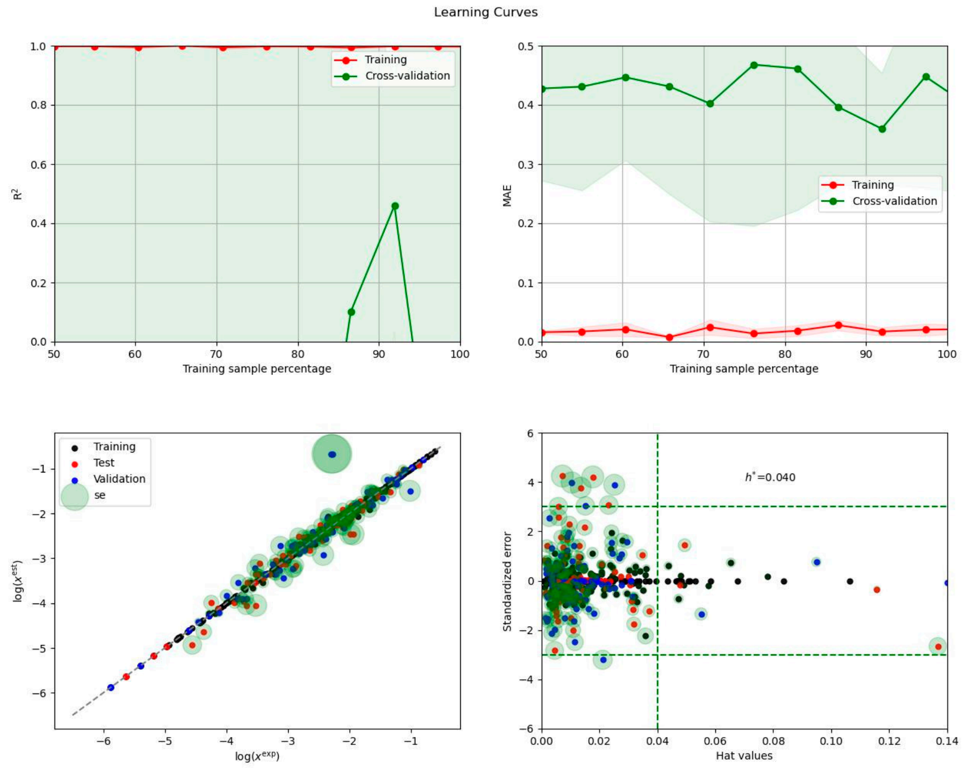

Figure S11. The graphical representation of the performance of ExtraTreeRegressor. The meaning of the panels is the same as in Figure S2.1.
